# Genetic variation in *PLEKHG1* is associated with white matter hyperintensities (n = 11,226)

**DOI:** 10.1212/WNL.0000000000006952

**Published:** 2019-02-19

**Authors:** Matthew Traylor, Daniel J. Tozer, Iain D. Croall, Danuta M. Lisiecka Ford, Abiodun Olubunmi Olorunda, Giorgio Boncoraglio, Martin Dichgans, Robin Lemmens, Jonathan Rosand, Natalia S. Rost, Peter M. Rothwell, Cathie L.M. Sudlow, Vincent Thijs, Loes Rutten-Jacobs, Hugh S. Markus

**Affiliations:** From the Department of Clinical Neurosciences, Stroke Research Group (M.T., D.J.T., I.D.C., D.M.L.F., A.O.O., L.R.-J., H.S.M.), University of Cambridge, UK; Department of Cerebrovascular Diseases (G.B.), Fondazione IRCCS Istituto Neurologico “Carlo Besta,” Milan, Italy; Institute for Stroke and Dementia Research (M.D.), Klinikum der Universität München, Ludwig-Maximilians-Universität München, Munich; German Center for Neurodegenerative Diseases (DZNE) and Munich Cluster for Systems Neurology (SyNergy) (M.D.), Germany; Department of Neurosciences, Experimental Neurology and Leuven Research Institute for Neuroscience and Disease (LIND) (R.L.), KU Leuven–University of Leuven; Department of Neurology (R.L.), University Hospitals Leuven; Laboratory of Neurobiology (R.L.), VIB Center for Brain and Disease Research, Leuven, Belgium; Center for Human Genetic Research (J.R.) and Division of Neurocritical Care and Emergency Neurology (J.R.) and J. Philip Kistler Stroke Research Center (J.R., N.S.R.), Department of Neurology, Massachusetts General Hospital, Boston; Nuffield Department of Clinical Neurosciences (Clinical Neurology), Stroke Prevention Research Unit (P.M.R.), University of Oxford; Centre for Clinical Brain Sciences and Institute for Genetics and Molecular Medicine (C.L.M.S.), University of Edinburgh, UK; Stroke Division, Florey Institute of Neuroscience and Mental Health (V.T.), University of Melbourne; and Department of Neurology (V.T.), Austin Health, Heidelberg, Victoria, Australia.

## Abstract

**Objective:**

To identify novel genetic associations with white matter hyperintensities (WMH).

**Methods:**

We performed a genome-wide association meta-analysis of WMH volumes in 11,226 individuals, including 8,429 population-based individuals from UK Biobank and 2,797 stroke patients. Replication of novel loci was performed in an independent dataset of 1,202 individuals. In all studies, WMH were quantified using validated automated or semi-automated methods. Imputation was to either the Haplotype Reference Consortium or 1,000 Genomes Phase 3 panels.

**Results:**

We identified a locus at genome-wide significance in an intron of *PLEKHG1* (rs275350, β [SE] = 0.071 [0.013]; *p* = 1.6 × 10^−8^), a Rho guanine nucleotide exchange factor that is involved in reorientation of cells in the vascular endothelium. This association was validated in an independent sample (overall *p* value, 2.4 × 10^−9^). The same single nucleotide polymorphism was associated with all ischemic stroke (odds ratio [OR] [95% confidence interval (CI)] 1.07 [1.03–1.12], *p* = 0.00051), most strongly with the small vessel subtype (OR [95% CI] 1.09 [1.00–1.19], *p* = 0.044). Previous associations at 17q25 and 2p16 reached genome-wide significance in this analysis (rs3744020; β [SE] = 0.106 [0.016]; *p* = 1.2 × 10^−11^ and rs7596872; β [SE] = 0.143 [0.021]; *p* = 3.4 × 10^−12^). All identified associations with WMH to date explained 1.16% of the trait variance in UK Biobank, equivalent to 6.4% of the narrow-sense heritability.

**Conclusions:**

Genetic variation in *PLEKHG1* is associated with WMH and ischemic stroke, most strongly with the small vessel subtype, suggesting it acts by promoting small vessel arteriopathy.

Cerebral white matter hyperintensities (WMH) are a radiologic marker of cerebral small vessel disease (SVD), the major cause of vascular dementia, and the pathology underlying both subcortical lacunar ischemic strokes and deep intracerebral hemorrhages (ICH), which together constitute a significant proportion of stroke. Furthermore, WMH are associated with both stroke and dementia risk in the general population.^1^ Despite the clinical importance of WMH, their pathogenesis is incompletely understood. Vascular risk factors, such as elevated blood pressure and diabetes mellitus, are thought to be important and management of these factors is currently the only treatment for SVD.

Identifying new treatments for SVD is constrained by our limited understanding of the underlying disease. Genetic studies are one of the few ways of identifying genuinely new pathways leading to disease and therefore hold promise to discover new potential treatments. Twin studies have demonstrated a high heritability for WMH,^2,3^ suggesting genetic factors are likely to confer considerable disease risk. Previous genome-wide association studies (GWAS) have uncovered a number of loci that influence WMH,^4,5^ and these in combination have been shown in turn to increase risk of lacunar stroke.^6^ However, these variants explain only a fraction of disease risk, motivating further identification of WMH-associated loci.

In this analysis, we performed a GWAS of WMH in 11,226 individuals to identify novel genetic variants associated with WMH.

## Methods

### Study populations and ethical approval

Two study populations were combined in a meta-analysis to examine the genetic basis of WMH: UK Biobank and the WMH in Stroke Study. A third population was used for replication: the Massachusetts General Hospital (MGH) WMH Study.

UK Biobank (ukbiobank.ac.uk) is a prospective study that recruited 500,000 community-dwelling participants aged 40–69 years from across the United Kingdom between 2006 and 2010. The study collects extensive data from questionnaires, interviews, health records, physical measures, biological samples, and imaging.

A subset of UK Biobank participants also underwent MRI of the head.^7^ This study used the second release of MRI data, including 9,045 participants who underwent brain MRI, on average 6.6 years (SD 1.0 years) after initial recruitment at mean age 55.5 years (SD 7.4 years) and had usable T2 fluid-attenuated inversion recovery (FLAIR) or diffusion tensor imaging images. We excluded participants with a diagnosis of stroke (ICD-9/ICD-10 or self-report or health record linkage), multiple sclerosis, Parkinson disease, dementia, or any other neurodegenerative disease at baseline, as well as participants with no genetic data. Following visual inspection of the data by the authors, 3 further participants with outlying tract-averaged water diffusion biomarker values were removed.

The WMH in Stroke Study data have been described previously.^5^ Ischemic stroke populations were enrolled between 1995 and 2013 through hospital-based studies. Further data are available from Open Science Framework (table e-1, osf.io/pkruv). We excluded from analyses patients with cerebral autosomal dominant arteriopathy with subcortical infarcts and leukoencephalopathy (CADASIL) or any other suspected monogenic cause of stroke, vasculitis, or any other nonischemic cause of WMH such as demyelinating and mitochondrial disorders.

Data were derived from contributing studies of the Wellcome Trust Case Control Consortium 2 (WTCCC2) (Oxford, Edinburgh, Munich, and St. George's, University of London [SGUL]), as well as other hospital-based cohorts: Milano Besta, Genes and Ischaemic Stroke (GENESIS), Leuven Stroke Study, South London Ethnicity and Stroke Study (SLESS), and DNA Lacunar.

The MGH WMH Study data have been described previously.^5^ MRI scans were derived from MGH, Ischemic Stroke Genetics Study, Australian Stroke Genetics Collaborative studies, and the Siblings with Ischemic Stroke Study.

### Genotype data

The UK Biobank genotyping procedure has been described elsewhere.^8^ In short, 2 custom genotyping arrays were used to genotype 49,950 participants (UK BiLEVE Axiom Array) and 438,427 participants (UK Biobank Axiom Array).^8,9^ Genotype data (805,426 markers) were available for 488,377 individuals, and were subsequently imputed to the Haplotype Reference Consortium (HRC) reference panel (39,131,578 autosomal single nucleotide polymorphisms [SNPs]). Imputed genotypes were available for 487,442 individuals in this study.^8^ From the resulting imputed dataset, we excluded (1) individuals who did not segregate with European individuals based on principal component analysis, (2) individuals with high levels of heterozygosity or missingness (>5%), and (3) individuals whose reported sex did not match with sex inferred from the genetic data. Only SNPs imputed from the HRC panel were included in this analysis.

The WMH in Stroke Study data genotyping has been described previously.^5^ All data were genotyped on commercially available Illumina arrays. Quality control procedures involved removing SNPs and individuals with high levels of missingness, discordant phenotypic and genotypic sex, strand ambiguous SNPs, and deviations from Hardy-Weinberg equilibrium (*p* < 1 × 10^−6^). For this analysis, all datasets were then imputed to 1,000 Genomes phase 3 (all populations) using SHAPEIT2 and IMPUTE2.

The MGH WMH Study data genotyping has been described previously.^5^ All individuals were genotyped on commercially available GWAS arrays from Affymetrix (Santa Clara, CA) or Illumina (San Diego, CA). Standard quality control was performed on the data, equivalent to the WMH in Stroke Study, and the datasets were imputed to 1,000 Genomes phase 1 integrated variant set using SHAPEIT2 and IMPUTE2.

### Standard protocol approvals, registrations, and patient consents

UK Biobank received ethical approval from the research ethics committee (reference 11/NW/0382). All participants provided informed consent to participate. The present analyses were conducted under UK Biobank application number 36509. An institutional review board or regional review board has approved the use of human participants in each of the WMH in Stroke study populations. All patients gave informed consent.

### Phenotype derivation

UK Biobank procedures for brain imaging acquisition are available on the UK Biobank website (Brain Imaging Documentation V1.3, ukbiobank.ac.uk). Our analyses made use of imaging-derived phenotypes/preprocessed image data estimated by an image-processing pipeline developed and run on behalf of UK Biobank.^10^ In brief, all brain MRI data were acquired on one standard Siemens Skyra 3T scanner (Siemens Medical Solutions, Munich, Germany) using the standard Siemens 32-channel RF receiver head coil. T1-weighted sagittal scans were acquired using a 3D magnetization-prepared rapid acquisition gradient echo sequence (resolution 1 × 1 × 1 mm, field of view 208 × 256 × 256, inversion time [TI]/repetition time [TR] = 880/2,000 ms). T2-weighted FLAIR sagittal scans were obtained using a 3D SPACE sequence (resolution 1.05 × 1.0 × 1.0 mm, field of view 192 × 256 × 256, TI/TR = 1,800/5,000 ms). Details of quality control procedures for the MRI data and image processing are freely available on the UK Biobank website (Brain Imaging Documentation V1.3, ukbiobank.ac.uk) and have been previously described. WMH were segmented automatically using the combined T1 and T2 FLAIR data as input in the Brain Intensity Abnormality Classification Algorithm (BIANCA) tool.^11^ BIANCA is a fully automated supervised method for WMH detection that gives the probability per voxel of being WMH based on a k-nearest neighbor algorithm. The total WMH volume was calculated from the voxels within a white matter mask exceeding a probability of 0.9 of being WMH. Subsequent values were corrected for natural head size based on the total intracranial volume and natural log transformed due to their skewed distribution.

In the WMH in Stroke Study data, we largely employed the same methodology as in our previous analysis.^5^ MRI scans were acquired as part of routine clinical practice for evaluation of ischemic stroke. FLAIR sequences were primarily used for WMH volumetric analysis; however, in their absence, T2-weighted sequences were used (WTCCC2-Oxford and WTCCC2-Munich only). In all scans, to avoid confounding by hyperintense signal due to the stroke for which the patient was enrolled, WMH were assessed in the contralateral hemisphere to the acute event. Lacunar infarcts were identified as low signal on T1 or FLAIR images using standard criteria and were excluded from WMH estimates.^12^ Trained raters blinded to all patient information outside of the MRI analyzed anonymized MRI scans. All supratentorial white matter and deep gray matter lesions were included in WMH volumes with the exception of WMH corresponding to infarcts, both lacunar and territorial.^13^ MRI scans with excessive movement artefact, incomplete brain coverage, or bihemispheric infarcts (other than lacunar) were excluded.

The WTCCC2, GENESIS (1 and 2), SGUL, Leuven, and Milan cohorts were analyzed using DISPunc, a semiautomated lesion drawing software.^14^ A manually marked “seed” at the lesion border was first identified, and then outlined automatically based on the signal intensity gradient. Visual inspection and manual correction of each WMH region of interest (ROI) was then performed as required. The SLESS and GENESIS 3 datasets were analyzed separately. WMH were defined as areas of increased signal on FLAIR images and segmented using a semi-automated contouring technique in Jim image analysis software version 7.0 5 (Xinapse Systems Limited; xinapse.com/j-im-7-software/) and corrected as necessary. To estimate total intracranial volume (TICV) in all WMH in Stroke Study datasets, T2-weighted and, in their absence, FLAIR sequences were analyzed using an automated segmentation program: SIENAX, part of FSL,^15^ which calculates the total volume of CSF and gray and white matter volumes. For all participants, WMH volumes were doubled to obtain a whole brain estimate and then corrected for TICV, thereby correcting for natural differences in head size. The values were subsequently natural log transformed.

For the MGH WMH Study, the protocol matched that of the WMH in Stroke Study above. FLAIR sequences were analyzed using a semi-automated method using MRIcro as previously described.^13^ Overlapping ROIs corresponding to WMH were marked to produce the final maps for WMH volume calculation. Intracranial area (ICA) was derived as the average of 2 midsagittal slices traced based on anatomical landmarks on T1 sequences, and used to correct for natural differences in head size.^16^ The phenotype for analysis was derived as above.

### Genome-wide analysis, meta-analysis, and replication

The UK Biobank and WMH in Stroke Study populations were analyzed separately and then combined in a meta-analysis.

In UK Biobank, we performed a GWAS of log (WMH), using SNPTEST v2.5.4-beta3. We included age at MRI, sex, genotyping batch, and the first 10 ancestry informative principal components as covariates. Data contributing to the WMH in Stroke datasets were analyzed using plink v1.90b3.45, including age at MRI, sex, and ancestry informative principal components as covariates.

Before performing a meta-analysis, we evaluated the degree of genetic correlation between the 2 datasets using LDSCORE.^17^ After confirming high genetic correlation, we performed a joint analysis of the datasets, meta-analyzing using metal.^18^ SNPs with *p* values for association with WMH less than 5 × 10^−8^ were considered significant.

For SNPs reaching *p* < 5 × 10^−8^, we sought replication in the MGH WMH Study. We evaluated the overall evidence for association by performing an inverse variance weighted meta-analysis.

All studies corrected for either intracranial volume or ICA in derivation of the phenotype (see above) to correct for natural differences in head size.

Genomic control (GC) correction was applied to all individual studies.^19^ Our primary analysis did not include GC correction of the overall meta-analysis (double-GC correction) as this is considered conservative.^17,20^ However, for completeness, we did perform a secondary double-GC correction of meta-analysis *p* values, which we include in the Results.

### Fine-mapping derived from credible SNP set analyses

We calculated credible SNP sets for loci associated with WMH at genome-wide significance to identify the set of SNPs that contain the causal SNP with 95% certainty. We calculated Bayes factors from the effect sizes and standard errors using Wakefield^21^ approximation for all SNPs in linkage disequilibrium (*r*^2^ > 0.1) with the lead SNP. Based on these Bayes factors, we calculated the posterior probability that each specific variant is causal, as well as the 95% credible set for each association (the smallest set of variants with posteriors that sum to at least 95%).^22^

### Association of *PLEKHG1* SNP with related neurologic and cardiovascular traits

For a novel SNP (rs275350) associated with WMH, we examined associations with related neurologic and cardiovascular traits, namely ischemic stroke and its subtypes; cardioembolic, large vessel, and small vessel stroke^23^; ICH (including subtypes by location: deep and lobar)^24^; coronary artery disease^25^; and Alzheimer disease.^26^ We note that rs275350 did not pass quality control in the ICH datasets, so a close proxy SNP (rs12202497, *r*^2^ = 0.93) was used in its place. Further data are available from Open Science Framework (table e-2; osf.io/pkruv).

### Bioinformatics analyses of a *PLEKHG1* locus using publicly available databases

To identify the putative function of a novel locus, we interrogated a variety of online resources. We identified the tissue-specific expression pattern of the gene in which the novel SNP resides using GTEx portal (gtexportal.org).^27^ Using the same resource, we tested whether the lead SNP was associated with expression of the same gene in brain, vascular, and blood tissues. We used the Capture HiC Plotter (chicp.org/), which interrogates data pertaining to interactions between distant DNA elements and enhancer and promoter sites of genes, to assess interactions between our *PLEKHG1* locus and genome-wide genes.^28,29^ We used the International Mouse Phenotyping Consortium data (mousephenotype.org/) to evaluate the phenotype of mice with *PLEKHG1* knocked out.^30^ We used PhenoScanner (phenoscanner.medschl.cam.ac.uk/phenoscanner) to identify any published associations between the *PLEKHG1* variant and proxies (*r*^2^ > 0.6) and any GWAS, eQTL, or metabolite phenotype.^31^

### Data availability

Full summary statistics from the meta-analysis are available to download from the Cerebrovascular Portal (cerebrovascularportal.org/informational/downloads).

## Results

### Genome-wide meta-analysis

After quality control, 8,429 individuals were analyzed in UK Biobank, and 2,797 in the WMH in Stroke dataset. The genetic correlation between the 2 datasets was very high (rG [SE] = 0.93 [0.68]), indicating high sharing of genetic mechanisms between the 2 populations, and justifying combining the 2 populations in a meta-analysis. Genomic inflation factors (λ) and LDSCORE intercepts indicated no significant inflation in the WMH in stroke (λ = 1.04, intercept = 1.04) and UK Biobank datasets (λ = 1.05, intercept = 1.01), as well as for the resulting meta-analyzed dataset (λ = 1.05, intercept = 1.01).^17,19^ QQ plots data are available from Open Science Framework (figures e-1 to e-4; osf.io/pkruv).

In the genome-wide meta-analysis of the 11,226 participants, we identified an association intronic of the *PLEKHG1* gene (rs275350; β [SE] = 0.071 [0.013]; *p* = 1.6 × 10^−8^). The SNP was associated with WMH in both UK Biobank (β [SE] = 0.065 [0.013]; *p* = 7.9 × 10^−7^) and in stroke populations (β [SE] = 0.12 [0.041]; *p* = 0.0026). We also confirmed 2 previous associations at genome-wide significance: one at 17q25, localizing to *TRIM47* and *TRIM65* (rs3744020; β [SE] = 0.106 [0.016]; *p* = 1.2 × 10^−11^), and a second on chromosome 2, close to *EFEMP1* (rs7596872; β [SE] = 0.143 [0.021]; *p* = 3.4 × 10^−12^).^4,5^ For all 3 loci, there was no evidence of heterogeneity either within the WMH in Stroke cohorts or between the WMH in Stroke and UK Biobank datasets (table; forest plots data available from Open Science Framework [figures e-5 and e-6; osf.io/pkruv]). Of the 12 SNPs previously reported as being associated with WMH,^4,5^ all had consistent direction of effect in this analysis, and 9 had *p* < 0.05 (further data available from Open Science Framework [table e-2; osf.io/pkruv]). All 13 SNPs associated with WMH at genome-wide significance to date explained 1.16% of the WMH variance in UK Biobank, of which rs275350 contributed 0.19%. Using LDSCORE, we estimated heritability of WMH to be 18.0%, meaning these 13 variants explain 6.4% of the narrow-sense heritability, of which rs275350 contributed 1.1%.

**Table:**
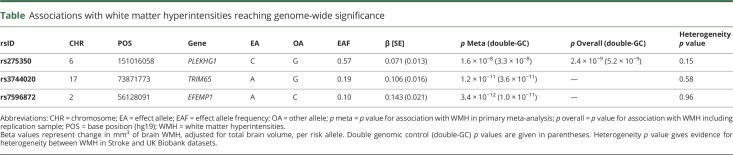
Associations with white matter hyperintensities reaching genome-wide significance

We sought replication of the *PLEKHG1* SNP in a further 1,202 stroke patients from the MGH WMH study. There was a consistent effect in this population (β [SE] = 0.080 [0.042]; *p* = 0.057), increasing evidence for association of the SNP overall (*p* = 2.5 × 10^−9^).

Credible sets for the *PLEKHG1* locus contained 6 SNPs (rs275350, rs6940540, rs12192990, rs62434144, rs12202497, rs6916149); 3 SNPs for the *EFEMP1* locus (rs7596872, rs146896516, rs113481311); and 33 SNPs for the 17q25 locus.

### Association of rs275350 with related neurologic and cardiovascular traits

**Figure 1:**
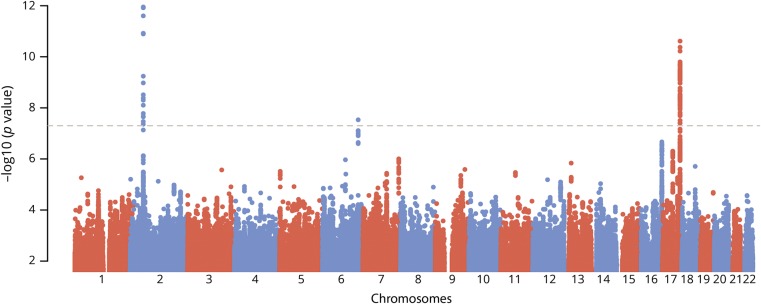
Manhattan plot of genome-wide –log 10 (*p* values) for association with white matter hyperintensities by genomic position

**Figure 2:**
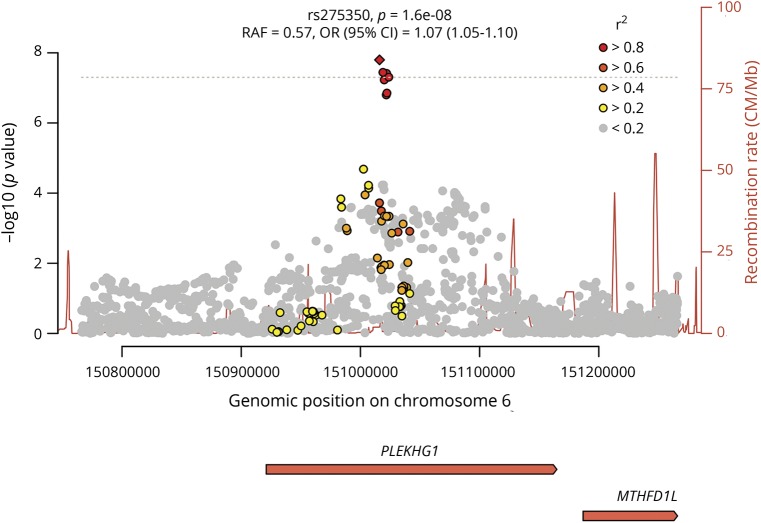
Local plot shows –log 10 (*p* values) for associations with white matter hyperintensities at *PLEKHG1* locus Linkage disequilibrium *r*^2^ with lead single nucleotide polymorphism (SNP) given in shades of red, orange, and yellow. SNPs with low *r*^2^ with the lead SNP (*r*^2^ < 0.2) are shown in gray. CI = confidence interval; OR = odds ratio; *PLEKHG1* = pleckstrin homology and Rho guanine nucleotide exchange factor domain containing G1; *MTHFD1L* = methylenetetrahydrofolate dehydrogenase (NADP + dependent) 1 like gene; RAF = risk allele frequency.

**Figure 3:**
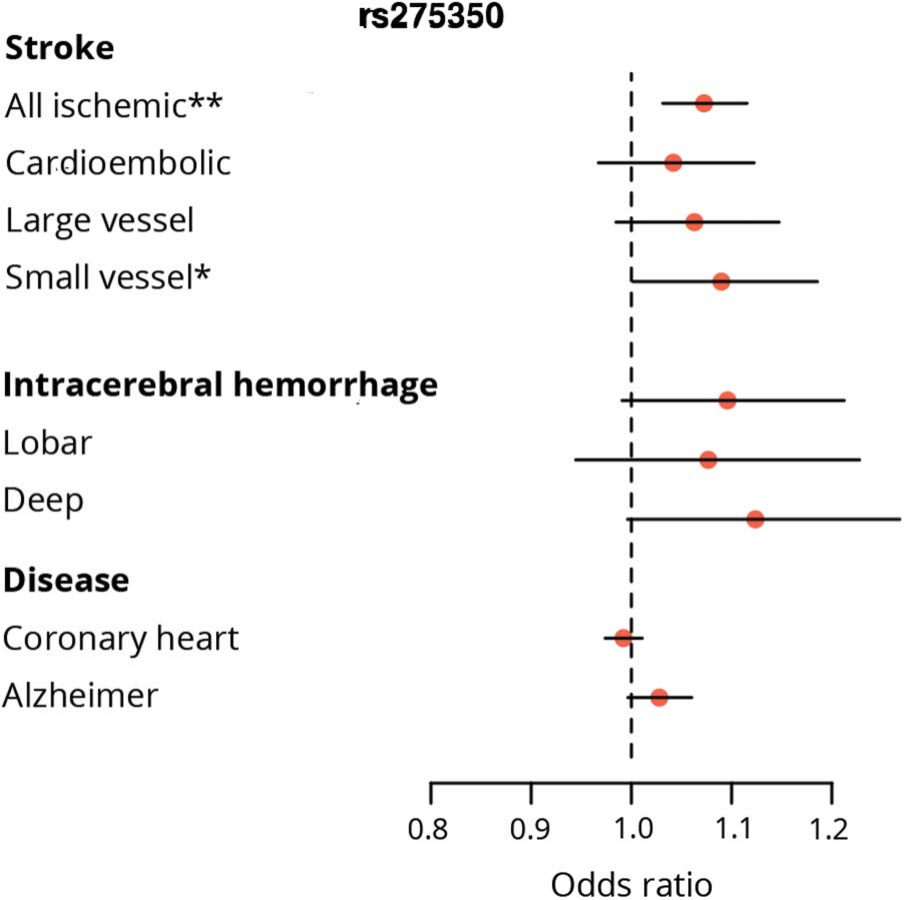
Association of rs275350 with related neurologic and cardiovascular traits

The *PLEKHG1* SNP (rs275350) was associated with all ischemic stroke (odds ratio [OR] [95% confidence interval (CI)] 1.07 [1.03–1.12], *p* = 0.00051; figures 1–3), most strongly with the small vessel subtype (OR [95% CI] 1.09 [1.00–1.19], *p* = 0.044). The effects for ICH were similar to small vessel stroke (OR [95% CI] 1.10 [0.99–1.21], *p* = 0.075), particularly for deep ICH (OR [95% CI] 1.12 [1.00–1.27], *p* = 0.058), but were nonsignificant due to larger CIs. Conversely, there was no evidence of association with coronary heart disease or Alzheimer disease.

### Bioinformatics analyses of rs275350 using publicly available databases

*PLEKHG1* is widely expressed in many tissues, including brain and arterial tissues (further data available from Open Science Framework [figure e-7]; osf.io/pkruv). Capture Hi-C data showed interactions between DNA at rs275350 with promoter sites of *PLEKHG1* as well as nearby genes *ZBTB2* and *PPP1R14C* in GM12878 lymphoblastoid cells, suggesting the variant might influence expression of these genes. There is no evidence that *PLEKHG1* knockout mice have brain or cardiovascular defects, but female *PLEKHG1* knockout mice have elevated fructosamine levels (*p* = 1.2 × 10^−7^; sex interaction *p* = 1.0 × 10^−4^). We used GTEx portal to assess the relationship between rs275350 and *PLEKHG1* mRNA expression in brain, vascular, and blood tissues. There were nominally significant associations with expression of *PLEKHG1* in brain and vascular tissues (further data available from Open Science Framework [table e-4]; osf.io/pkruv), but none reached Bonferroni-corrected significance. A hypothesis-free search of the GTEx database highlighted an association with expression of PPP1R14C in esophageal mucosa (*p* = 1.9 × 10^−5^). Using PhenoScanner, we identified no other associations of rs275350, or proxies with *r*^2^ > 0.6, with any reported GWAS, eQTL, or metabolomic phenotypes.

## Discussion

In this genome-wide meta-analysis in 11,226 individuals, we identified an association with WMH in an intron of pleckstrin homology and Rho guanine nucleotide exchange factor (Rho-GEF) domain containing G1 (*PLEKHG1*), which we validated in an independent population. *PLEKHG1* belongs to a family of Rho-GEFs, which activate Rho family small GTPases by catalyzing the exchange of GDP for GTP. In the vascular endothelium, Rho-GEFs—including PLEKHG1—are involved in reorientation of cells and their stress fibers in response to mechanical stress.^32^ Disruption of this reorientation is a potential mechanism by which the identified variant might increase WMH burden. Variants in *PLEKHG1* have been associated with blood pressure in African Americans.^33,34^ However, the SNPs identified in this study were only weakly correlated with the blood pressure–associated variants (*r*^2^ = 0.057), suggesting the mechanism associated with the variant identified in this analysis is likely to be distinct. The SNP was also associated with all ischemic stroke, an association that was strongest for the small vessel subtype. Effects were similar for ICH, particularly subcortical, but were not significant. This association with other stroke phenotypes, particularly small vessel stroke, might point to the SNP increasing risk by a small vessel arteriopathy.

We explored whether the SNP in *PLEKHG1* is an eQTL, influencing expression of nearby genes, particularly *PLEKHG1*. There were nominal associations with *PLEKHG1* expression in brain and vascular tissues and an association with *PPP1R14C* in tissue presumed not to be disease relevant. Further interrogation of gene expression associations with genetic variation at the locus in disease relevant tissue will help to shed light on whether these are true-positive associations. From the data available, we are not able to determine which SNP is the causal variant that increases WMH burden. Novel methods such as massively parallel reporter assays may help to shed light on this.^35^ Furthermore, determining the biological function of GWAS-associated variants, such as the *PLEKHG1* variants identified here, remains challenging. However, notable recent successes have involved CRISPR-editing stem cell–derived cells,^36^ and such an approach has appeal for interrogating cerebral SVD-associated loci. In addition, recall-by-genotype and phenome-wide association studies, particularly with detailed MRI variables, have the potential to illuminate the full phenotypic spectrum of the associated variant.^37,38^

In addition, we validated 2 previously identified loci on chromosomes 2p16 (*EFEMP1*) and 17q25 (*TRIM47/TRIM65*). *EFEMP1* is an extracellular matrix glycoprotein, the gene product of which is fibulin 3. Fibulin 3 has been shown to have an antagonistic effect on vessel development and repressive effect on expression of some matrix metalloproteinases.^39^ Perhaps most interestingly, fibulin has been shown to induce expression of *TIMP3*, which ameliorates disease manifestations in CADASIL mice,^40^ highlighting shared pathologic processes in sporadic and monogenic small vessel disease. The 17q25 locus overlaps several genes, including 2 tripartite-containing motifs (*TRIM47*, *TRIM65*). As has been reported before,^4^ several of the genome-wide significant SNPs in this locus are missense variants, and many influence expression of *TRIM47* in brain tissues. We note that one SNP in the region, rs4600514 (p.Arg187Trp), in an exon of *TRIM47*, is predicted as “probably damaging” by Polyphen, “deleterious” by SIFT, and has a CADD score of 34,^41^ meaning it is in the top 0.1% of SNPs for likelihood of deleteriousness. This might point to knockout of *TRIM47* being the underlying mechanism conferring risk of WMH.

Our study has several strengths. The study had a large sample size. In UK Biobank, a uniform high-quality image acquisition protocol was used. Automated or semiautomatic segmentation methods were used to quantify WMH in all studies, which have benefits over ratings scale, which can be subject to ceiling effects. The *PLEKHG1* GWAS association we identified was validated in an independent population, although this did not quite reach statistical significance independently. However, there are also limitations. At this point, our observations cannot be generalized to non-European populations. Only 2% of participants of the UK Biobank imaging substudy were of non-European ancestry and were therefore excluded in this analysis. In the WMH in Stroke Study population, a small subset of scans were T2-weighted rather than FLAIR images, which can be less accurate for identifying WMH. In addition, methods for lesion and brain volume were different in the 2 groups. In the WMH in stroke population, a number of raters calculated lesion volumes. Interrater reliability was high (>0.9) among all individuals, but subtle differences and biases could still remain. To maximize sample size in this analysis, we combined data from community and stroke populations. We found high genetic correlation between the 2 populations and previous analyses have shown all associated genetic variants to influence disease risk in both populations.^5^ It is possible that including a population selected on the basis of disease could exert subtle influences on the estimated effect of genetic variants. However, there was no evidence of heterogeneity between the populations for any of the genome-wide significant variants and effects were observed in both cohorts. In age-related traits such as WMH, measurement of the phenotype in an aged population depends on survival of those individuals to the point of recruitment. Therefore, in addition, subtle survival biases may influence the estimated effect. As with all genome-wide studies, the most accurate estimate of the effect of a given variant should be obtained from a large independent population-based cohort.

Genetic variation in *PLEKHG1* is associated with WMH, as well as ischemic stroke and in particular small vessel stroke, suggesting it confers risk via small vessel arteriopathy.

## Glossary

BIANCA: Brain Intensity Abnormality Classification Algorithm
CADASIL: cerebral autosomal dominant arteriopathy with subcortical infarcts and leukoencephalopathy
CI: confidence interval
FLAIR: fluid-attenuated inversion recovery
GC: genomic control
GENESIS: Genes and Ischaemic Stroke
GWAS: genome-wide association studies
HRC: Haplotype Reference Consortium
ICA: intracranial area
ICD-9: International Classification of Diseases–9
ICD-10: International Classification of Diseases–10
ICH: intracerebral hemorrhage
MGH: Massachusetts General Hospital
OR: odds ratio
*PLEKHG1*: pleckstrin homology and Rho guanine nucleotide exchange factor domain containing G1
Rho-GEF: Rho guanine nucleotide exchange factor
ROI: region of interest
SGUL: St. George's, University of London
SLESS: South London Ethnicity and Stroke Study
SNP: single nucleotide polymorphism
SVD: small vessel disease
TI: inversion time
TICV: total intracranial volume
TR: repetition time
WMH: white matter hyperintensities
WTCCC2: Wellcome Trust Case Control Consortium 2
